# Crystal structure of *cis*-1-(2-methyl-1,2,3,4-tetra­hydro­quinolin-4-yl)azepan-2-one

**DOI:** 10.1107/S1600536814017826

**Published:** 2014-08-09

**Authors:** P. S. Pradeep, S. Naveen, M. N. Kumara, K. M. Mahadevan, N. K. Lokanath

**Affiliations:** a Department of Chemistry, Kuvempu University, Jnanasahyadri, Shankaraghatta 577 451, India; b Institution of Excellence, University of Mysore, Manasagangotri, Mysore 570 006, India; c Department of Chemistry, Yuvaraja’s College, University of Mysore, Mysore 570 005, India; d Department of Studies in Physics, University of Mysore, Manasagangotri, Mysore 570 006, India

**Keywords:** crystal structure, tetra­hydro­quinolines, azepan-2-one, hydrogen bonding

## Abstract

In the title compound, C_16_H_22_N_2_O, the azepan-2-one ring adopts a chair conformation, while the 1,2,3,4-tetra­hydro­pyridine ring adopts a half-chair conformation. In the crystal, mol­ecules are linked by N—H⋯O hydrogen bonds, forming supra­molecular chains propagated along [10-1], with weak C—H⋯O inter­actions occurring between the chains.

## Related literature   

For applications of tetra­hydro­quinolines, see: Konishi *et al.* (1990 ▶).
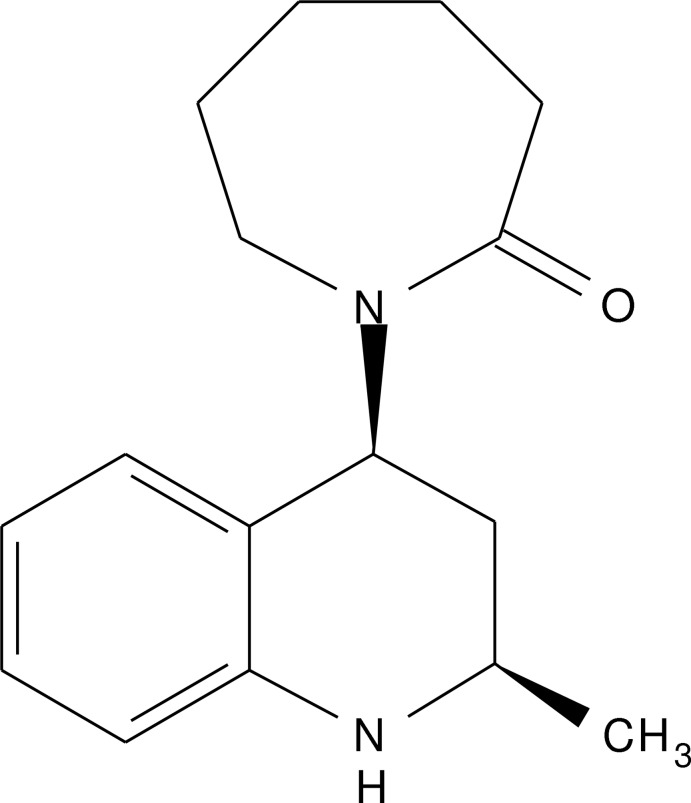


## Experimental   

### Crystal data  

C_16_H_22_N_2_O
*M* *_r_* = 258.36
Monoclinic, 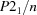

*a* = 9.1640 (17) Å
*b* = 13.1687 (18) Å
*c* = 11.988 (2) Å
β = 96.825 (11)°
*V* = 1436.4 (4) Å^3^
*Z* = 4
Cu *K*α radiation
μ = 0.59 mm^−1^
*T* = 296 K
0.23 × 0.22 × 0.21 mm

### Data collection  

Bruker X8 Proteum diffractometer
Absorption correction: multi-scan (*SADABS*; Bruker, 2013 ▶) *T*_min_ = 0.874, *T*_max_ = 0.884
7903 measured reflections
2343 independent reflections
2106 reflections with *I* > 2σ(*I*)
*R*_int_ = 0.028

### Refinement  

*R*[*F* ^2^ > 2σ(*F* ^2^)] = 0.049
*wR*(*F* ^2^) = 0.139
*S* = 1.06
2343 reflections
173 parameters
H-atom parameters constrained
Δρ_max_ = 0.34 e Å^−3^
Δρ_min_ = −0.36 e Å^−3^

### 

Data collection: *APEX2* (Bruker, 2013 ▶); cell refinement: *SAINT* (Bruker, 2013 ▶); data reduction: *SAINT*; program(s) used to solve structure: *SHELXS97* (Sheldrick, 2008 ▶); program(s) used to refine structure: *SHELXL97* (Sheldrick, 2008 ▶); molecular graphics: *Mercury* (Macrae *et al.*, 2008 ▶); software used to prepare material for publication: *Mercury*.

## Supplementary Material

Crystal structure: contains datablock(s) global, I. DOI: 10.1107/S1600536814017826/xu5808sup1.cif

Structure factors: contains datablock(s) I. DOI: 10.1107/S1600536814017826/xu5808Isup2.hkl

Supporting information file. DOI: 10.1107/S1600536814017826/xu5808Isup3.cml

**Fig. 1.**
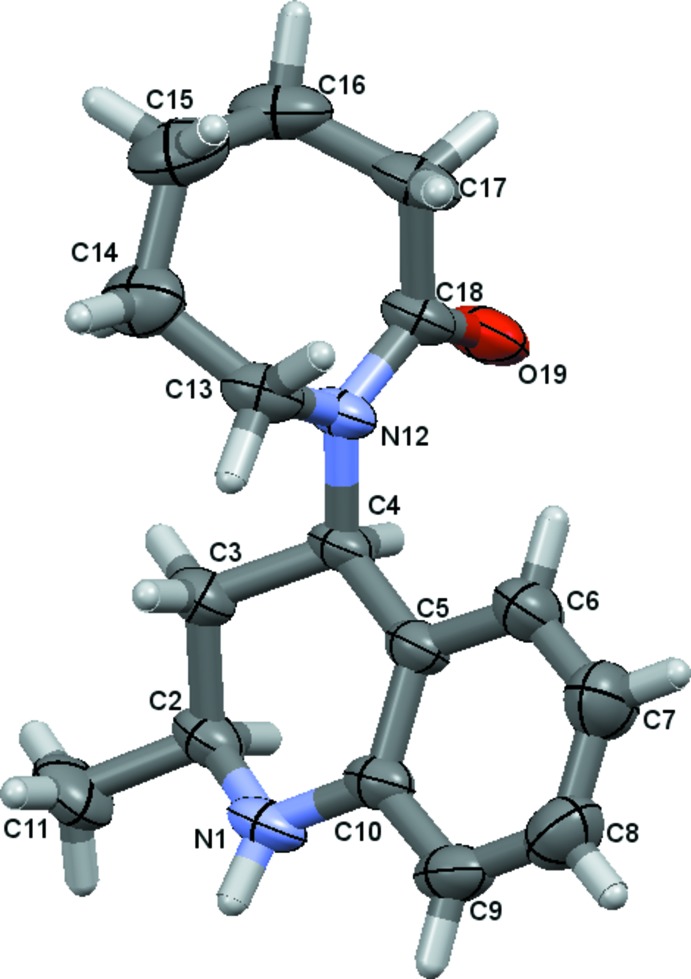
A view of the title molecule, with atom labelling. Displacement ellipsoids are drawn at the 50% probability level.

**Fig. 2.**
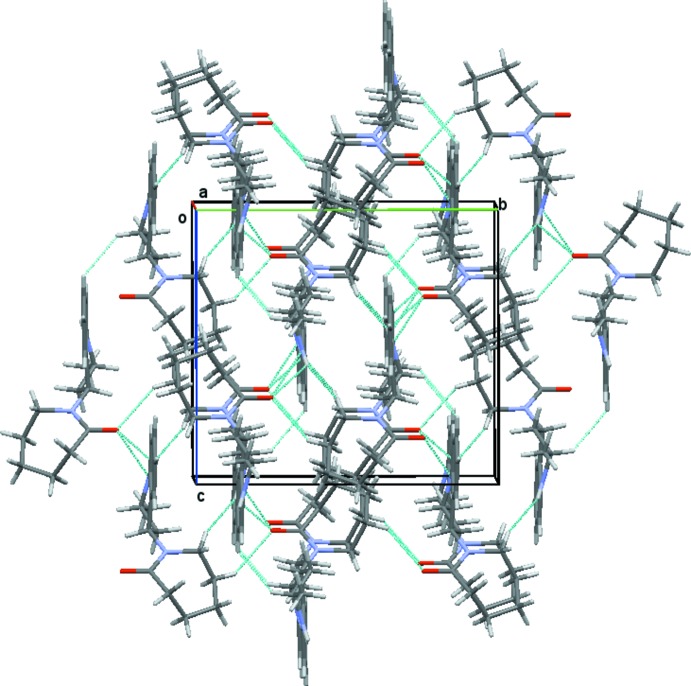
A viewed along the a axis of the crystal packing of the title compound.

. DOI: 10.1107/S1600536814017826/xu5808fig1.tif
A view of the title mol­ecule, with atom labelling. Displacement ellipsoids are drawn at the 50% probability level.

a . DOI: 10.1107/S1600536814017826/xu5808fig2.tif
A viewed along the *a* axis of the crystal packing of the title compound.

CCDC reference: 1017682

Additional supporting information:  crystallographic information; 3D view; checkCIF report

## Figures and Tables

**Table 1:** Hydrogen-bond geometry (Å, °)

*D*—H⋯*A*	*D*—H	H⋯*A*	*D*⋯*A*	*D*—H⋯*A*
N1—H1⋯O19^i^	0.86	2.40	2.988 (2)	126
C14—H14*A*⋯O19^ii^	0.97	2.57	3.320 (2)	134

Symmetry codes: (i) 
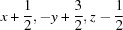
; (ii) 
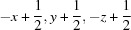
.

## supplementary crystallographic information

### S1. Comment

Tetrahydroquinolines are an important family of heterocyclic compounds having wide range of biological activities which includes antimalarial, antitumoral, antioxidant, *etc*. In particular 2-Methyl-1,2,3,4-tetrahydroquinoline is present in human brain and a natural antitumor antibiotic, has a complex structure built on the tetrahydroquinoline system (Konishi *et al.*, 1990). Hence, in continuation of our effort to identify new quinoline based therapeutic agents, the title compound has been synthesized and herein we report its crystal structure.

The *ORTEP* of the molecule is shown in figure 1. The title compound is chiral. In the arbitrarily chosen asymmetric molecule, C2 has *S* configuration whereas C4 has *R* configuration. The azepan ring lies in the equatorial plane of the fused rings as indicated by the dihedral angle value of 66.28 (8)°. A study of torsion angles, asymmetric parameters and least squares plane calculations reveals that the quinoline ring in the structure adopts a half chair conformation with the atom C3 deviating by 0.3278 (18) Å from the least-squares plane defined by the atoms N1/C2/C3/C4/C5/C10. This is confirmed by the puckering amplitude *Q* = 0.4857 (18) Å. The structure exhibits weak intermolecular hydrogen bonds of the type C—H···O and N—H···O. The packing of the molecules when viewed along the *a* axis indicate that they are stacked in pairs.

### S2. Experimental

A catalytic amount of SbF_3_ (10 mol %) was added to the mixture of aniline(1 equivalent) and *N*-vinyl caprolactam(2–3 equivalent) in acetonitrile (5–10 ml). The reaction mixture was stirred at ambient temperature (~25 °C) for 20–70 min. The reaction was monitored by TLC by using ethyl acetate/hexane as eluent. After the completion of the reaction, the solvent was removed under *vacuo*. The crude product was then quenched with water and the catalyst was decomposed by addition of appropriate amount of sodium bicarbonate solution, extracted with ethyl acetate (10 ml × 5 times), dried and was purified by column chromatography using ethyl acetate/hexane as eluent (pet ether/ethyl acetate 80:20 *v*/*v*). The white solid crystals were obtained by slow evaporation method by using petroleum ether: ethyl acetate, 8:2 *v*/*v* as solvents. M.P. = 155–160 °C. Yield: 93%.

### S3. Refinement

The hydrogen atoms were placed geometrically with N—H = 0.86, C—H = 0.93–0.98 Å, and allowed to ride on their parent atoms with *U*_iso_(H) = 1.5*U*_eq_(C) for the methyl H atoms and 1.2*U*_eq_(N,C) for the others.

### Crystal data

**Table:**

C_16_H_22_N_2_O	*F*(000) = 560
*M**_r_* = 258.36	*D*_x_ = 1.195 Mg m^−^^3^
Monoclinic, *P*2_1_/*n*	Cu *K*α radiation, λ = 1.54178 Å
Hall symbol: -P 2yn	Cell parameters from 2343 reflections
*a* = 9.1640 (17) Å	θ = 6.7–64.8°
*b* = 13.1687 (18) Å	µ = 0.59 mm^−^^1^
*c* = 11.988 (2) Å	*T* = 296 K
β = 96.825 (11)°	Block, colorless
*V* = 1436.4 (4) Å^3^	0.23 × 0.22 × 0.21 mm
*Z* = 4	

### Data collection

**Table:**

Bruker X8 Proteum diffractometer	2343 independent reflections
Radiation source: Bruker MicroStar microfocus rotating anode	2106 reflections with *I* > 2σ(*I*)
Helios multilayer optics monochromator	*R*_int_ = 0.028
Detector resolution: 18.4 pixels mm^-1^	θ_max_ = 64.8°, θ_min_ = 6.7°
φ and ω scans	*h* = −10→10
Absorption correction: multi-scan (*SADABS*; Bruker, 2013)	*k* = −15→4
*T*_min_ = 0.874, *T*_max_ = 0.884	*l* = −14→13
7903 measured reflections	

### Refinement

**Table:**

Refinement on *F*^2^	Primary atom site location: structure-invariant direct methods
Least-squares matrix: full	Secondary atom site location: difference Fourier map
*R*[*F*^2^ > 2σ(*F*^2^)] = 0.049	Hydrogen site location: inferred from neighbouring sites
*wR*(*F*^2^) = 0.139	H-atom parameters constrained
*S* = 1.06	*w* = 1/[σ^2^(*F*_o_^2^) + (0.0671*P*)^2^ + 0.4283*P*] where *P* = (*F*_o_^2^ + 2*F*_c_^2^)/3
2343 reflections	(Δ/σ)_max_ < 0.001
173 parameters	Δρ_max_ = 0.34 e Å^−^^3^
0 restraints	Δρ_min_ = −0.36 e Å^−^^3^

### Special details

**Table:**

Geometry. Bond distances, angles *etc*. have been calculated using the rounded fractional coordinates. All su's are estimated from the variances of the (full) variance-covariance matrix. The cell e.s.d.'s are taken into account in the estimation of distances, angles and torsion angles
Refinement. Refinement on *F*^2^ for ALL reflections except those flagged by the user for potential systematic errors. Weighted *R*-factors *wR* and all goodnesses of fit *S* are based on *F*^2^, conventional *R*-factors *R* are based on *F*, with *F* set to zero for negative *F*^2^. The observed criterion of *F*^2^ > σ(*F*^2^) is used only for calculating -*R*-factor-obs *etc*. and is not relevant to the choice of reflections for refinement. *R*-factors based on *F*^2^ are statistically about twice as large as those based on *F*, and *R*-factors based on ALL data will be even larger.

### Fractional atomic coordinates and isotropic or equivalent isotropic displacement parameters (Å^2^)

**Table:**

	*x*	*y*	*z*	*U*_iso_*/*U*_eq_	
O19	0.20029 (15)	0.75502 (10)	0.32245 (11)	0.0661 (5)	
N1	0.53573 (16)	0.85016 (11)	−0.01036 (12)	0.0538 (5)	
N12	0.28345 (14)	0.90157 (10)	0.25474 (10)	0.0438 (4)	
C2	0.60480 (18)	0.82217 (14)	0.10008 (15)	0.0511 (6)	
C3	0.52791 (17)	0.87701 (14)	0.18769 (14)	0.0497 (5)	
C4	0.36664 (17)	0.84597 (12)	0.17675 (13)	0.0421 (5)	
C5	0.29631 (17)	0.85491 (12)	0.05626 (13)	0.0439 (5)	
C6	0.1450 (2)	0.86076 (16)	0.03030 (16)	0.0593 (6)	
C7	0.0790 (2)	0.86425 (19)	−0.07861 (17)	0.0718 (8)	
C8	0.1649 (3)	0.86264 (17)	−0.16454 (16)	0.0700 (7)	
C9	0.3153 (2)	0.85678 (14)	−0.14226 (15)	0.0605 (7)	
C10	0.38380 (19)	0.85289 (12)	−0.03180 (14)	0.0459 (5)	
C11	0.7676 (2)	0.84612 (19)	0.1097 (2)	0.0743 (8)	
C13	0.28372 (19)	1.01269 (13)	0.25122 (14)	0.0514 (6)	
C14	0.3621 (2)	1.06237 (15)	0.35552 (17)	0.0640 (7)	
C15	0.2718 (3)	1.06735 (17)	0.45383 (18)	0.0770 (8)	
C16	0.2169 (3)	0.96542 (17)	0.49058 (17)	0.0720 (8)	
C17	0.1190 (2)	0.90829 (16)	0.40028 (17)	0.0627 (7)	
C18	0.20364 (18)	0.84820 (14)	0.32180 (13)	0.0486 (6)	
H1	0.58880	0.86510	−0.06260	0.0640*	
H2	0.59290	0.74890	0.10990	0.0610*	
H3A	0.53550	0.94980	0.17750	0.0600*	
H3B	0.57490	0.86010	0.26220	0.0600*	
H4	0.36380	0.77400	0.19710	0.0510*	
H6	0.08640	0.86240	0.08850	0.0710*	
H7	−0.02280	0.86770	−0.09380	0.0860*	
H8	0.12120	0.86550	−0.23860	0.0840*	
H9	0.37220	0.85540	−0.20150	0.0730*	
H11A	0.81160	0.80920	0.05330	0.1120*	
H11B	0.81330	0.82670	0.18280	0.1120*	
H11C	0.78080	0.91760	0.09920	0.1120*	
H13A	0.18280	1.03640	0.24060	0.0620*	
H13B	0.33020	1.03440	0.18670	0.0620*	
H14A	0.38960	1.13080	0.33670	0.0770*	
H14B	0.45190	1.02500	0.37870	0.0770*	
H15A	0.33110	1.09830	0.51720	0.0920*	
H15B	0.18770	1.11120	0.43340	0.0920*	
H16A	0.16270	0.97610	0.55430	0.0860*	
H16B	0.30120	0.92320	0.51580	0.0860*	
H17A	0.05640	0.86230	0.43620	0.0750*	
H17B	0.05600	0.95660	0.35650	0.0750*	

### Atomic displacement parameters (Å^2^)

**Table:**

	*U*^11^	*U*^22^	*U*^33^	*U*^12^	*U*^13^	*U*^23^
O19	0.0806 (9)	0.0577 (9)	0.0692 (9)	−0.0120 (7)	0.0477 (7)	−0.0006 (6)
N1	0.0525 (8)	0.0624 (9)	0.0526 (8)	0.0030 (7)	0.0321 (7)	0.0032 (7)
N12	0.0447 (7)	0.0501 (8)	0.0406 (7)	0.0023 (6)	0.0221 (6)	0.0023 (6)
C2	0.0446 (9)	0.0534 (10)	0.0599 (10)	0.0014 (7)	0.0254 (8)	0.0009 (8)
C3	0.0411 (8)	0.0607 (10)	0.0501 (9)	−0.0022 (7)	0.0173 (7)	−0.0045 (8)
C4	0.0419 (8)	0.0472 (9)	0.0412 (8)	−0.0003 (7)	0.0212 (6)	0.0003 (7)
C5	0.0441 (9)	0.0469 (9)	0.0436 (9)	−0.0039 (7)	0.0179 (7)	−0.0008 (7)
C6	0.0468 (10)	0.0805 (13)	0.0529 (10)	−0.0060 (9)	0.0161 (8)	0.0002 (9)
C7	0.0567 (11)	0.0964 (16)	0.0609 (12)	−0.0073 (11)	0.0017 (9)	0.0001 (11)
C8	0.0819 (14)	0.0791 (14)	0.0470 (10)	−0.0082 (11)	0.0000 (10)	−0.0047 (9)
C9	0.0812 (14)	0.0618 (11)	0.0424 (9)	−0.0068 (9)	0.0238 (9)	−0.0057 (8)
C10	0.0555 (9)	0.0415 (9)	0.0448 (9)	−0.0023 (7)	0.0227 (7)	−0.0012 (7)
C11	0.0451 (10)	0.0932 (16)	0.0900 (15)	0.0016 (10)	0.0301 (10)	−0.0044 (12)
C13	0.0576 (10)	0.0523 (10)	0.0480 (9)	0.0097 (8)	0.0219 (8)	0.0091 (7)
C14	0.0803 (13)	0.0496 (11)	0.0641 (12)	0.0029 (9)	0.0170 (10)	−0.0019 (9)
C15	0.1127 (18)	0.0636 (13)	0.0579 (12)	0.0138 (12)	0.0233 (12)	−0.0091 (10)
C16	0.0985 (16)	0.0738 (14)	0.0498 (11)	0.0185 (12)	0.0348 (10)	−0.0001 (9)
C17	0.0607 (11)	0.0735 (13)	0.0616 (11)	0.0111 (9)	0.0388 (9)	0.0080 (10)
C18	0.0454 (9)	0.0591 (11)	0.0454 (9)	0.0002 (7)	0.0225 (7)	0.0031 (7)

### Geometric parameters (Å, º)

**Table:**

O19—C18	1.228 (2)	C2—H2	0.9800
N1—C2	1.446 (2)	C3—H3A	0.9700
N1—C10	1.386 (2)	C3—H3B	0.9700
N12—C4	1.470 (2)	C4—H4	0.9800
N12—C13	1.464 (2)	C6—H6	0.9300
N12—C18	1.347 (2)	C7—H7	0.9300
N1—H1	0.8600	C8—H8	0.9300
C2—C11	1.516 (3)	C9—H9	0.9300
C2—C3	1.516 (2)	C11—H11A	0.9600
C3—C4	1.524 (2)	C11—H11B	0.9600
C4—C5	1.515 (2)	C11—H11C	0.9600
C5—C6	1.387 (2)	C13—H13A	0.9700
C5—C10	1.400 (2)	C13—H13B	0.9700
C6—C7	1.373 (3)	C14—H14A	0.9700
C7—C8	1.369 (3)	C14—H14B	0.9700
C8—C9	1.375 (3)	C15—H15A	0.9700
C9—C10	1.397 (2)	C15—H15B	0.9700
C13—C14	1.515 (3)	C16—H16A	0.9700
C14—C15	1.520 (3)	C16—H16B	0.9700
C15—C16	1.517 (3)	C17—H17A	0.9700
C16—C17	1.521 (3)	C17—H17B	0.9700
C17—C18	1.512 (3)		
			
C2—N1—C10	119.82 (14)	C5—C4—H4	107.00
C4—N12—C13	118.48 (12)	C5—C6—H6	119.00
C4—N12—C18	118.62 (13)	C7—C6—H6	119.00
C13—N12—C18	122.79 (14)	C6—C7—H7	120.00
C2—N1—H1	120.00	C8—C7—H7	120.00
C10—N1—H1	120.00	C7—C8—H8	120.00
C3—C2—C11	112.49 (16)	C9—C8—H8	120.00
N1—C2—C3	108.91 (14)	C8—C9—H9	120.00
N1—C2—C11	109.66 (16)	C10—C9—H9	120.00
C2—C3—C4	109.79 (14)	C2—C11—H11A	109.00
N12—C4—C5	111.95 (13)	C2—C11—H11B	109.00
C3—C4—C5	111.08 (13)	C2—C11—H11C	109.00
N12—C4—C3	112.50 (13)	H11A—C11—H11B	110.00
C6—C5—C10	118.62 (15)	H11A—C11—H11C	110.00
C4—C5—C6	121.27 (15)	H11B—C11—H11C	109.00
C4—C5—C10	120.05 (14)	N12—C13—H13A	109.00
C5—C6—C7	122.08 (17)	N12—C13—H13B	109.00
C6—C7—C8	119.17 (19)	C14—C13—H13A	109.00
C7—C8—C9	120.51 (18)	C14—C13—H13B	109.00
C8—C9—C10	120.89 (17)	H13A—C13—H13B	108.00
N1—C10—C5	120.90 (15)	C13—C14—H14A	109.00
N1—C10—C9	120.33 (16)	C13—C14—H14B	109.00
C5—C10—C9	118.75 (16)	C15—C14—H14A	109.00
N12—C13—C14	114.22 (14)	C15—C14—H14B	109.00
C13—C14—C15	114.23 (16)	H14A—C14—H14B	108.00
C14—C15—C16	114.64 (18)	C14—C15—H15A	109.00
C15—C16—C17	114.77 (18)	C14—C15—H15B	109.00
C16—C17—C18	113.52 (17)	C16—C15—H15A	109.00
N12—C18—C17	116.96 (16)	C16—C15—H15B	109.00
O19—C18—N12	122.76 (16)	H15A—C15—H15B	108.00
O19—C18—C17	120.27 (16)	C15—C16—H16A	109.00
N1—C2—H2	109.00	C15—C16—H16B	109.00
C3—C2—H2	109.00	C17—C16—H16A	109.00
C11—C2—H2	109.00	C17—C16—H16B	109.00
C2—C3—H3A	110.00	H16A—C16—H16B	108.00
C2—C3—H3B	110.00	C16—C17—H17A	109.00
C4—C3—H3A	110.00	C16—C17—H17B	109.00
C4—C3—H3B	110.00	C18—C17—H17A	109.00
H3A—C3—H3B	108.00	C18—C17—H17B	109.00
N12—C4—H4	107.00	H17A—C17—H17B	108.00
C3—C4—H4	107.00		
			
C10—N1—C2—C3	−44.6 (2)	C3—C4—C5—C6	−160.41 (16)
C10—N1—C2—C11	−168.08 (16)	C3—C4—C5—C10	22.4 (2)
C2—N1—C10—C5	16.6 (2)	C4—C5—C6—C7	−176.99 (19)
C2—N1—C10—C9	−165.36 (16)	C10—C5—C6—C7	0.2 (3)
C13—N12—C4—C3	56.10 (18)	C4—C5—C10—N1	−4.7 (2)
C13—N12—C4—C5	−69.81 (17)	C4—C5—C10—C9	177.21 (15)
C18—N12—C4—C3	−127.62 (15)	C6—C5—C10—N1	178.05 (16)
C18—N12—C4—C5	106.48 (16)	C6—C5—C10—C9	0.0 (2)
C4—N12—C13—C14	−113.04 (16)	C5—C6—C7—C8	−0.5 (3)
C18—N12—C13—C14	70.8 (2)	C6—C7—C8—C9	0.5 (3)
C4—N12—C18—O19	1.8 (2)	C7—C8—C9—C10	−0.3 (3)
C4—N12—C18—C17	−179.65 (14)	C8—C9—C10—N1	−178.00 (17)
C13—N12—C18—O19	177.93 (15)	C8—C9—C10—C5	0.1 (3)
C13—N12—C18—C17	−3.5 (2)	N12—C13—C14—C15	−79.6 (2)
N1—C2—C3—C4	60.77 (18)	C13—C14—C15—C16	56.7 (3)
C11—C2—C3—C4	−177.44 (16)	C14—C15—C16—C17	−59.5 (3)
C2—C3—C4—N12	−176.30 (13)	C15—C16—C17—C18	83.3 (2)
C2—C3—C4—C5	−49.92 (18)	C16—C17—C18—O19	112.4 (2)
N12—C4—C5—C6	−33.7 (2)	C16—C17—C18—N12	−66.2 (2)
N12—C4—C5—C10	149.10 (14)		

### Hydrogen-bond geometry (Å, º)

**Table:**

*D*—H···*A*	*D*—H	H···*A*	*D*···*A*	*D*—H···*A*
N1—H1···O19^i^	0.86	2.40	2.988 (2)	126
C14—H14*A*···O19^ii^	0.97	2.57	3.320 (2)	134

Symmetry codes: (i) *x*+1/2, −*y*+3/2, *z*−1/2; (ii) −*x*+1/2, *y*+1/2, −*z*+1/2.
