# Supplementary figures and images for: Crystal structure of cis-1-(2-methyl-1,2,3,4-tetra­hydro­quinolin-4-yl)azepan-2-one
Source: Acta Crystallogr Sect E Struct Rep Online. 2014 Aug 9;70(Pt 9):o981–2. doi: 10.1107/S1600536814017826 (PMC4186183; doi:10.1107/S1600536814017826)

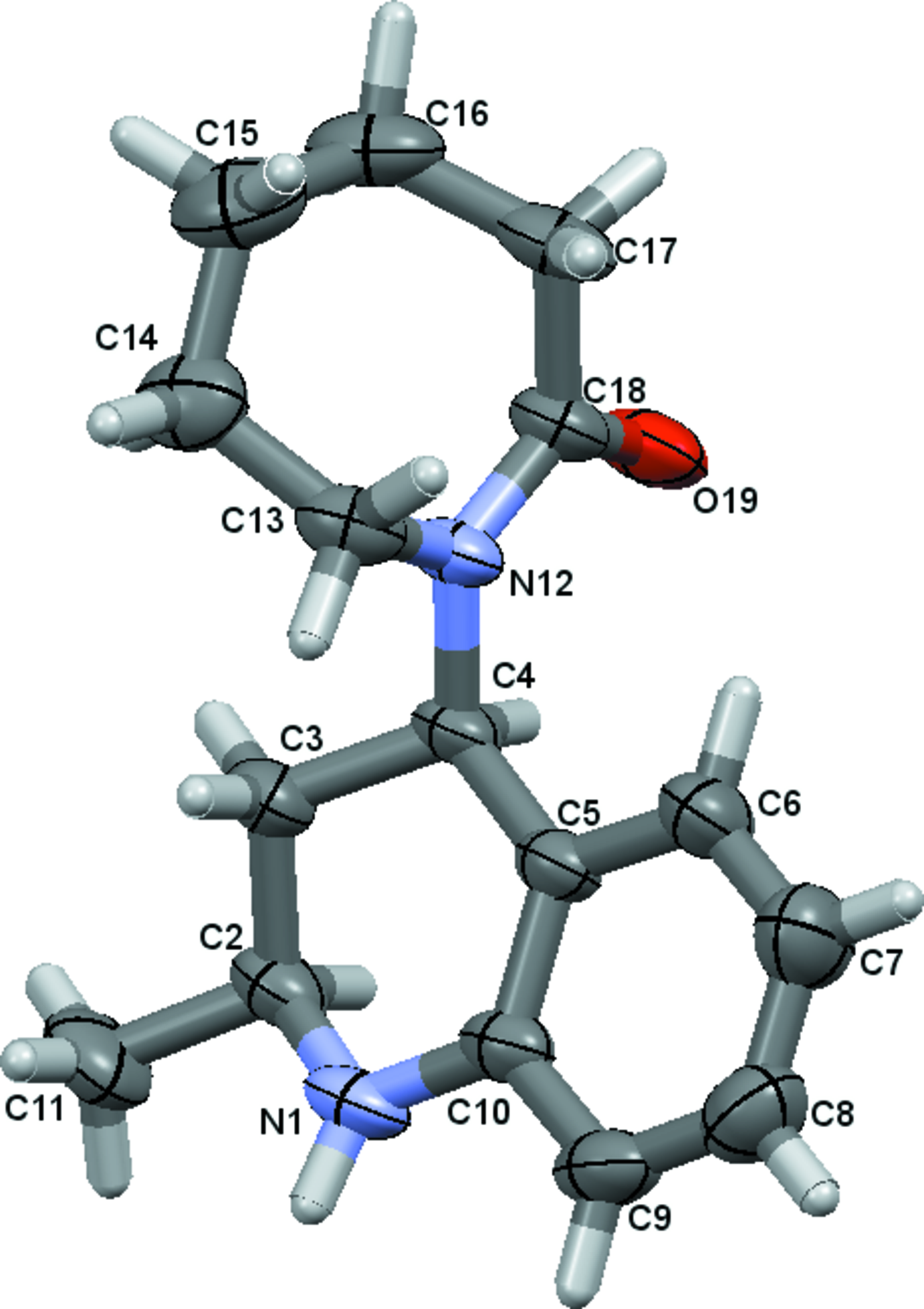

Supplement: Supplementary file 4 [file e-70-0o981-fig1.tif]

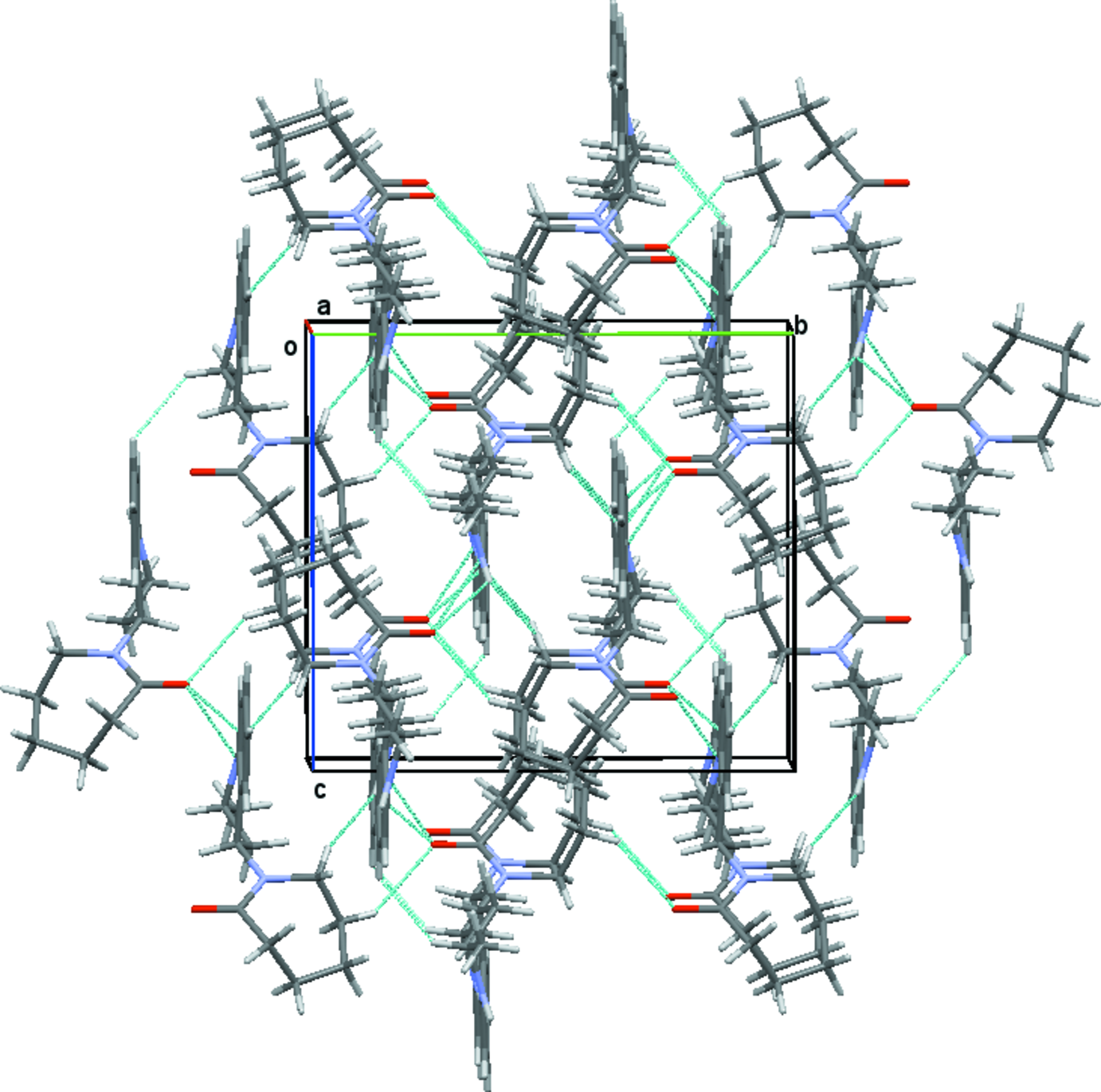

Supplement: Supplementary file 5 [file e-70-0o981-fig2.tif]
